# Concordance between biparametric MRI, transperineal targeted plus systematic MRI-ultrasound fusion prostate biopsy, and radical prostatectomy pathology

**DOI:** 10.1038/s41598-022-10672-4

**Published:** 2022-04-28

**Authors:** Tae Il Noh, Ji Sung Shim, Sung Gu Kang, Jun Cheon, Jeong Gu Lee, Jeong Hyeon Lee, Seok Ho Kang

**Affiliations:** 1 Department of Urology, Anam Hospital, Korea University College of Medicine, 73 Goryeodae-ro, Seongbuk-gu, Seoul, 02841 Korea; 2 Department of Pathology, Anam Hospital, Korea University College of Medicine, Seoul, Korea

**Keywords:** Cancer, Prostate cancer

## Abstract

We aimed to confirm the reliability of the results of bi-parametric magnetic resolution imaging-ultrasound fusion targeted and systematic biopsies (bpMRI-US transperineal FTSB) compared to prostatectomy specimens. We retrospectively analyzed the records of 80 men who underwent bpMRI-US transperineal FTSB with region of interest (ROI) and subsequent robot-assisted radical prostatectomy. Changes in the grade group determined by MRI and biopsy versus surgical specimens were analyzed. Thirty-five patients with insignificant prostate cancer and 45 with significant cancer were diagnosed using bpMRI-US transperineal FTSB. Among those with insignificant PCa, 25 (71.4%) were upgraded to significant PCa in prostatectomy specimens: 9/12 (75.0%) with Prostate Imaging Reporting and Data System (PI-RADS) 3, 12/16 (75.0%) with PI-RADS 4, and 4/7 (57.1%) with PI-RADS 5. In the PI-RADS 3 group, the upgraded group showed higher prostate specific antigen (PSA) and PSA density (PSAD) than the concordance group; PSA 8.34(2.73) vs. 5.31(2.46) (*p* = 0.035) and PSAD 0.29(0.11) vs. 0.18(0.09) (*p* = 0.025). The results of prostate biopsy and prostatectomy specimens were inconsistent and underestimated in patients with MRI-visible lesions. Therefore, for precise and individualized treatment strategies for PCa with MRI-visible lesions, careful interpretation of biopsy result is required.

## Introduction

Depending on the aggressiveness of prostate cancer (PCa), the range of treatment strategies varies^1^. Active surveillance may be recommended for indolent low-risk localized PCa^2^. In contrast, radical prostatectomy, radiotherapy, androgen deprivation therapy (ADT), chemotherapy, or a combination of these modalities may be performed depending on risk classification^3^.

The risk classification and treatment strategies for PCa are determined according to clinical parameters such as prostate specific antigen (PSA), PSA density (PSAD), age, radiologic findings such as magnetic resonance imaging (MRI), and prostate biopsy findings^4^. In particular, prostate biopsy plays a key role in risk stratification for PCa^5^. However, the current gold standard transrectal 12-core systematic biopsy is associated with misdiagnosis or misclassification in over 30% (36.3%) of patients with PCa at the time of diagnosis compared to radical prostatectomy specimen^6^. The high rate of inconsistencies and inaccuracies with transrectal systematic biopsy is caused by uncertainty of index lesions and the multifocal nature of PCa^7,8^.

Given the limitations of transrectal biopsy, accumulating compelling evidence has shown that MRI can be used to increase the detection rate and accuracy of PCa diagnosis^9–11^. Thus, urologists are applying MRI for the diagnosis, risk classification and treatment of PCa^12–14^.

In the era of MRI for the diagnosis of PCa, targeted biopsy as an ideal form of PCa diagnosis could be considered to determine the risk stratification for PCa with high consistency and accuracy^15^. However, as targeted biopsy alone is associated with missed clinically significant PCa^16^, various guidelines suggest a combination of targeted and systematic biopsy for enhancing the diagnostic accuracy of prostate biopsy^17,18^.

However, there is a lack of research on the reliability of the risk classification from this combination of targeted and systematic biopsy when lesions suspicious for PCa are visible on MRI. Therefore, we aimed to investigate the reliability of PCa classification by combined targeted and systematic biopsies compared to radical prostatectomy specimen.

## Materials and methods

### Study design

From 2017 to 2020, we analyzed the medical records of 80 male patients with results of bpMRI-US transperineal FTSB and radical prostatectomy specimens.

Before prostate biopsy, bpMRI was performed on all men with suspected prostate cancer, including a raised PSA level (≥ 4.0 ng/mL) and/or abnormal findings on digital rectal examination. Accordingly, regions of interest (ROIs) were established on MRI, bpMRI-US transperineal FTSB were performed in 300 men.

Subsequent robot-assisted radical prostatectomy was performed in 80 patients with consideration of radiologic findings such as MRI, clinical parameters, and prostate biopsy results. Informed consent was obtained from patients after they received an explanation of the treatment options, such as active surveillance, radiation therapy, ADT, chemotherapy, and a combination of each modality.

### MRI protocol

**Figure 1:**
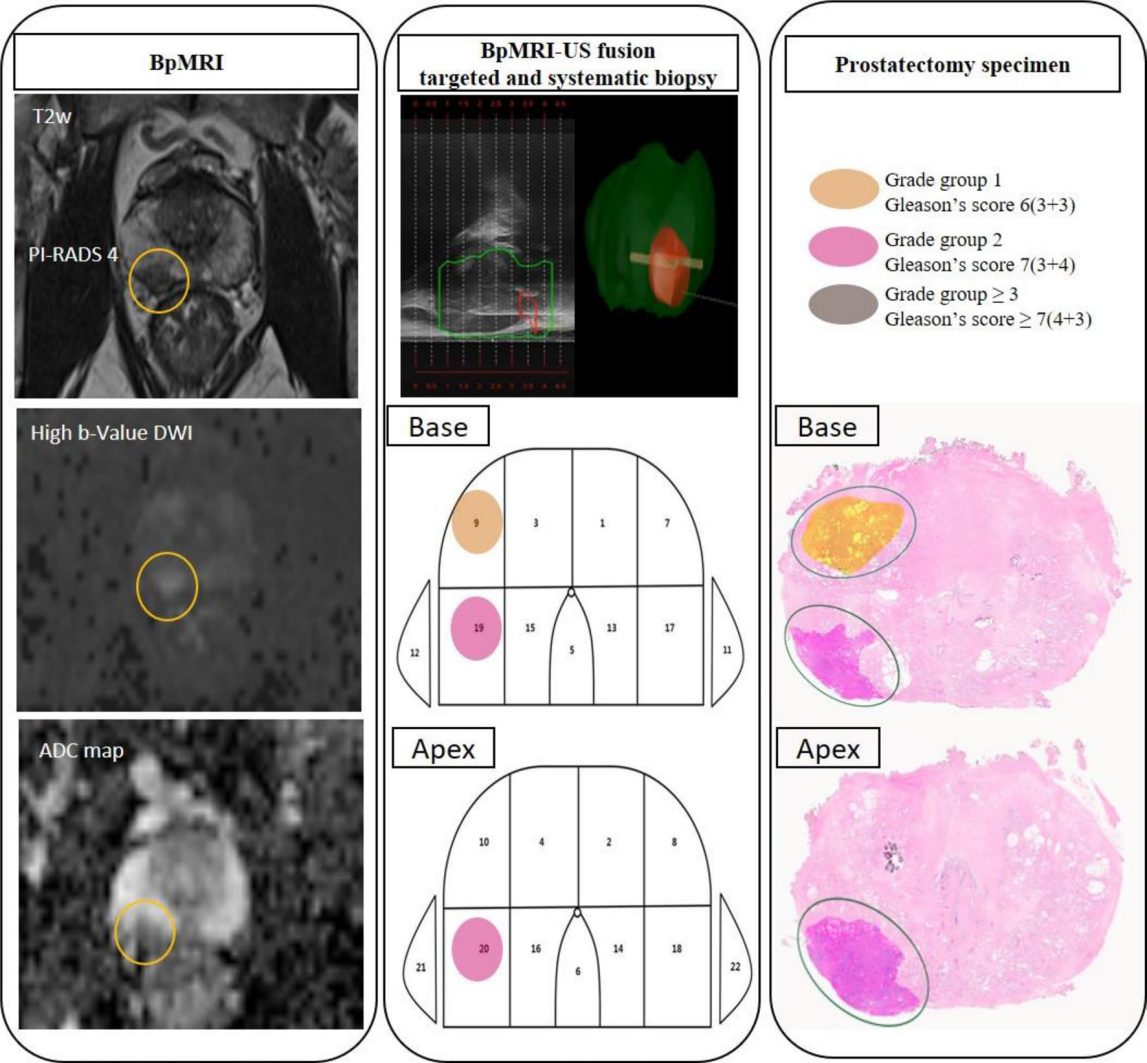
Comparison of MRI, prostate biopsy (targeted and systematic), and prostatectomy specimens.

Bi-parametric MRI was performed using a 3.0-T scanner (Siemens Medical System, Erlangen, Germany) without the dynamic contrast-enhanced imaging sequence from mpMRI. ROIs on bpMRI were marked by three dedicated uro-radiologists based on the Prostate Imaging- Reporting and Data System (PI-RADS), version 2.0. ROIs were set in areas with PI-RADS ≥ 3 on bpMRI and used as targeted regions (Fig. 1).

### Prostate biopsy protocol

We have previously reported a protocol for transperineal bpMRI-ultrasound fusion targeted and systematic biopsy^19^. In brief, the elastic image registration type of the MR-US fusion technique using a mechanical position encoder and robotic articulated arm system (Biojet, USA) was used, Targeted and systematic biopsies were performed in the same session. The number of biopsy cores was based on the prostate size as follows: 3–4 cores for targeted biopsy and 16–24 cores for systematic biopsy. The ROI lesion was not intentionally avoided during systematic biopsy, which was performed using a prostate mapping template (modified Barzell-template) in the routine manner of transperineal prostate biopsy. As shown in Fig. 1, 22-core systematic biopsy was performed depending on prostate size by template (1–22) (e.g., far distal section [11, 12, 21, 22] were omitted for small prostate sizes less than 20 cc; additional 2-core biopsies were performed in areas not covered by the template for prostate size larger than 60 cc) (Fig. 1).

### Histopathologic examination protocol

Whole-mount histopathology slides were used and each prostate was sectioned in the axial plane from the basal to the apex at approximately 4–5 mm intervals. All histopathologic examinations of the biopsy and radical prostatectomy specimens were reviewed by one uro-histopathologist to eliminate the inter-observer variability in grading the results of biopsies and radical prostatectomy specimens (Fig. 1).

### Definitions of terms

#### Region of interest

PI-RADS ≥ 3 on bpMRI as the ROI was marked by a uro-radiologist and used as targeted regions by urologists who performed prostate biopsy.

#### Clinically significant Pca

Clinically insignificant disease was defined as grade group 1 (Gleason score, 6 [3 + 3]). Clinically significant cancer was defined as more than grade group 2, which included Gleason grade pattern 4, grade group 2 (7 [3 + 4]), favorable intermediate risk and grade group 3 (7 [4 + 3]), and unfavorable intermediate risk.

#### Index lesion

The index lesion was defined as the largest prostatic carcinoma with the highest histologic grade, and was considered the most clinically significant tumor among the multifocal prostate tumors; the index lesion drives tumor behavior, growth, cellular proliferation, and progression.

### Ethics statement

This study was conducted according to the guidelines of the Declaration of Helsinki and the current ethical guidelines. The study was reviewed and approved by the Ethics Committee and the Institutional Review Board of Korea University Anam Hospital (IRB No. 2018AN0339). Written informed consent was obtained from all the study participants prior to their enrolment. 

## Results

The mean age of the included patients was 66.7 (8.4) years. The mean PSA level was 8.3 (6.5) ng/mL, and the mean PSAD was 0.27 (0.15) (Table 1).

**Table 1:** Patient characteristics.

Characteristics	Mean (SD or %)
Number of patients	80
Age, years	66.7 (8.4)
BMI, kg/m^2^	23.7 (5.2)
PSA, ng/mL	8.3 (6.5)
Prostate volume, mL	30.9 (16.1)
PSAD, ng/mL/g	0.27 (0.15)
**PI-RADS score, n (%)**	
3	20 (25.0)
4	33 (41.3)
5	27 (33.7)
Biopsy grade group *, n (%)	
1	35 (43.7)
2	15 (18.8)
≥ 3	30 (37.5)

*Biopsy grade groups: 1 = Gleason 6 (or less); 2 = Gleason 7 (3 + 4); 3 = Gleason 7 (4 + 3); 4 = Gleason 8; 5 = Gleason 9 or 10.

*BMI* Body mass index, *PSA* Prostate-specific antigen, *PI-RADS* Prostate Imaging Reporting and Data System.

### bpMRI-US transperineal FTSB

**Figure 2:**
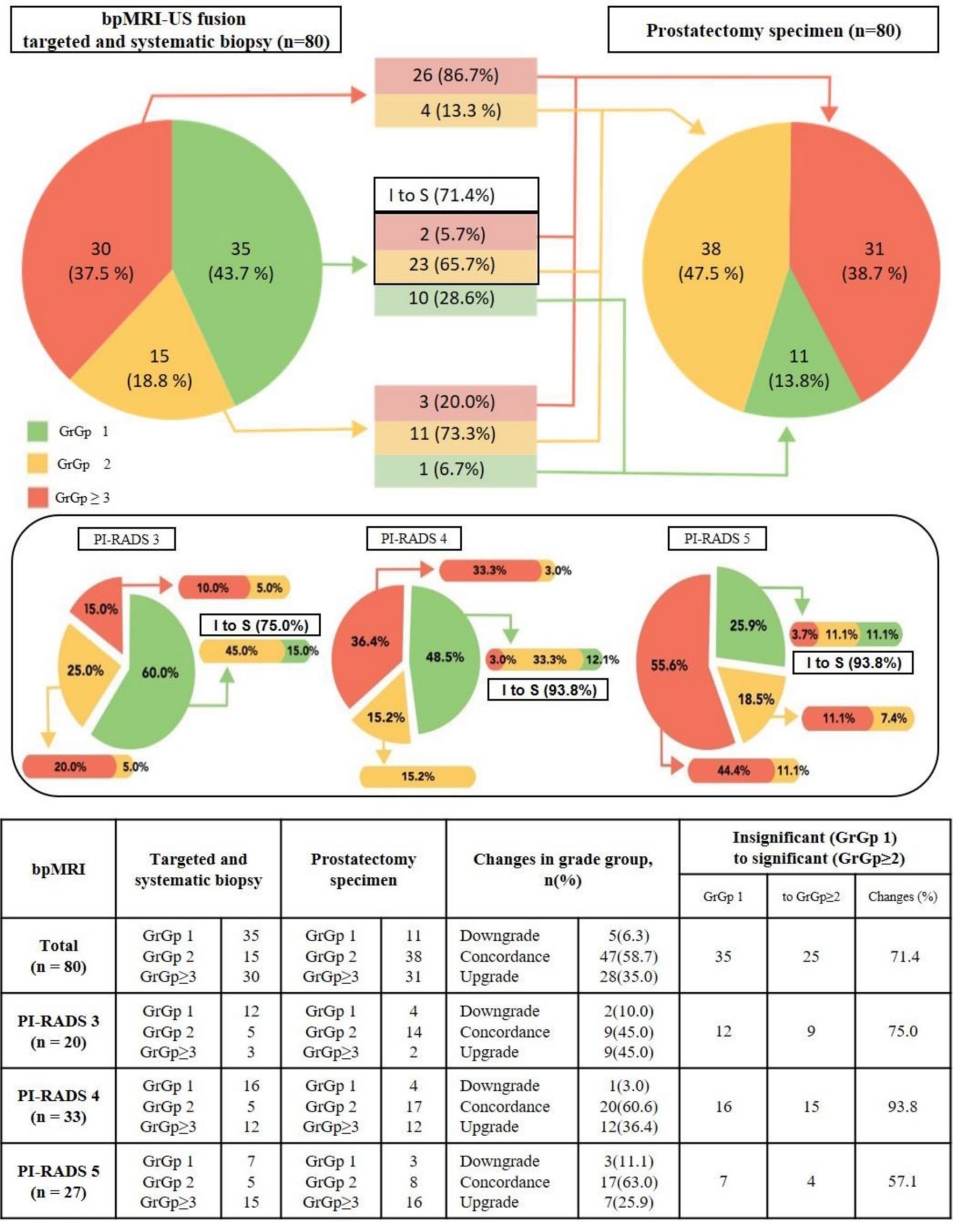
Cross-tabulation of grade group between prostate biopsy (targeted and systematic) and prostatectomy specimens. Biopsy grade groups: 1 = Gleason 6 (or less); 2 = Gleason 7 (3 + 4); 3 = Gleason 7 (4 + 3); 4 = Gleason 8; 5 = Gleason 9 or 10. insignificant prostate cancer (I) = GrGp 1; significant prostate cancer (S) = GrGp ≥ 2 bpMRI, bi-parametric magnetic resonance imaging; GrGp, grade group; PI-RADS, Prostate Imaging–Reporting and Data System.

Among the 80 men, 35 with GrGp1 (43.7%), 15 with GrGp2 (18.8%), and 30 with GrGp ≥ 3 (37.5%) were diagnosed using prostate biopsy. Thirty-five (43.7%) patients had clinically insignificant PCa (GrGp1): 12 of 20 (60.0%) with PI-RADS3, 16 of 33 (48.5%) with PI-RADS4, and 7 of 27 (25.9%) with PI-RADS5. Forty-five (56.2%) patients had clinically significant PCa (GrGp ≥ 2): 8 of 20 (40.0%) with PI-RADS3, 17 of 33 (51.5%) with PI-RADS4, and 20 of 27 (74.1%) with PI-RADS5 (Fig. 2).

### Radical prostatectomy specimen

#### Significant cancers

Clinically insignificant PCa (GrGp1) was found in 11 of 80 (13.8%) patients: 4 of 20 (20.0%) with PI-RADS3, 4 of 33 (12.1%) with PI-RADS4, and 3 of 27 (11.1%) with PI-RADS5. Clinically significant PCa (GrGp ≥ 2) was found in 69 of 80 (86.3%): 16 of 20 (80.0%) with PI-RADS3, 29 of 33 (87.9%) with PI-RADS4, and 24 of 27 (88.9%) with PI-RADS5 (Fig. 2).

#### Multifocality

Multifocality was observed in more than two multiple lesions in 65 of 80 (81.2%) patients, single lesions in 15 of 80 (18.8%), two lesions in 38 of 80 (33.8%), and more than three lesions in 27 of 80 (33.8%).

### Comparison between MRI and prostatectomy specimens

#### Significant cancers

There were 53.7% (43 of 80) of the patients with significant cancers in MRI-visible lesions: 7 of 20 (35.0%) with PI-RADS3, 16 of 33 (48.5%) with PI-RADS4, and 20 of 27 (74.1%) with PI-RADS5. Furthermore, 32.5% (26 of 80) had significant cancers in MRI-invisible lesions: 6 of 20 (30.0%) in PI-RADS3, 7 of 33 (21.2%) in PI-RADS4, and 13 of 27 (48.1%) in PI-RADS5 (Table 2).

**Table 2:** Index lesions and significant prostate cancers in MRI-visible and MRI-invisible lesions.

	N	MRI-visible lesions		MRI-invisible lesions	
		Significant cancers	Index lesion	Significant cancers	Index lesion
Total	80	43 (53.7%)	75 (93.7%)	26 (32.5%)	5 (6.3%)
PI-RADS 3	20 (25.0%)	7 (35.0%)	18 (90.0%)	6 (30.0%)	2 (10.0%)
PI-RADS 4	33 (41.3%)	16 (48.5%)	31 (93.9%)	7 (21.2%)	2 (6.1%)
PI-RADS 5	27 (33.7%)	20 (74.1%)	26 (96.3%)	13 (48.1%)	1 (3.7%)

*MRI* magnetic resonance imaging; *PI-RADS* Prostate Imaging–Reporting and Data System.

#### ndex lesions

Of all index lesions, 93.5% (75 of 80) were present in MRI-visible lesions and 6.5% (5 of 80) in MRI-invisible lesions (Table 2).

### Comparison between biopsy and prostatectomy specimens

#### Changes in GrGp between the biopsy and radical specimens

GrGp of biopsy compared to radical specimens showed concordance in 47 of 80 (58.7%), downgrading in 5 of 80 (6.3%), and upgrading in 28 of 80 (35.0%). A change from insignificant to significant cancer was observed in 25 of 35 (71.4%). GrGp1 by biopsy showed concordance with radical prostatectomy specimens in 10 of 35 (28.6%), and upgrading in 25 of 35 (71.4%). Specifically, in the PI-RADS 3 group, GrGp1 by biopsy showed concordance in 3 of 12 (25.0%), and upgrading in 9 of 12 (75.0%). The PI-RADS 4 group showed concordance in 4 of 16 (25.0%) patients, and upgrading in 12 of 16 (75.0%). The PI-RADS 5 group showed concordance in 3 of 7 (42.9%) patients,and upgrading in 4 of 7 (57.1%). (Fig. 2).

GrGp2 by biopsy showed concordance in 11 of 15 (73.3%), downgrading in 1 of 15 (6.7%), and upgrading in 3 of 15 (20.0%). Specifically, in the PI-RADS 3 group, concordance was observed in 4 of 5 (80.0%), downgrading in 1 of 5 (20.0%), and upgrading in 0 of 5 (0.0%). The PI-RADS 4 group showed concordance in 5 of 5 (100.0%) cases. In the PI-RADS 5 group, there was concordance in 2 of 5 (40.0%), downgrading in 0 of 5 (0.0%), and upgrading in 3 of 5 (60.0%) (Fig. 2).

GrGp ≥ 3 by biopsy showed concordance in 26 of 30 (86.7%) patients, downgrading in 4 of 30 (13.3%), and upgrading in 0 of 30 (0.0%). Specifically, in the PI-RADS 3 group, it showed concordance in 2 of 3 (66.7%), downgrading in 1 of 3 (33.3%), and upgrading in 0 of 3 (0.0%) patients. In the PI-RADS 4 group, concordance was observed in 11 of 12 (91.7%) patients, downgrading in 1 of 11 (8.3%), and upgrading in 0 of 12 (0.0%). In the PI-RADS 5 group, there was concordance in 12 of 15 (80.0%), downgrading in 3 of 15 (20.0%), and upgrading in 0 of 15 (0.0%) (Fig. 2).

There was no significant difference in clinical parameters among the three groups (downgrade, concordance, and upgrade) and in the group that changed from insignificant to significant cancer. However, specifically in the PI-RADS 3 group, the upgrade group showed higher PSA and PSAD than the concordance group. The PSA and PSAD values were as follows: upgrade vs. concordance group, PSA 8.34 (2.73) versus 5.31 (2.46) (*p* = 0.035); and PSAD 0.29 (0.11) versus 0.18 (0.09) (*p* = 0.025; Table 3).

**Table 3:** Comparison of clinical parameters according to changes in the grade group.

	Changes in grade group					Insignificant cancers in biopsy			
	Concordance (C)	Downgrade (D)	Upgrade (U)			Prostatectomy specimen			
						Insignificant (I)	Significant (S)		
N	27 (33.8%)	23 (28.7%)	30 (37.5%)	P^a^		10 (28.6%)	25 (71.4%)	P^b^	
				D vs U	C vs U			I vs. S	
Age	64.30 (6.04)	65.83 (6.83)	67.80 (5.69)	0.666	0.094	64.00 (5.27)	68.04 (5.69)	0.485	
PSA	7.39 (3.92)	7.01 (2.45)	13.74 (11.96)	0.018	0.934	5.56 (2.11)	6.27 (3.04)	0.436	
PV	31.52 (8.45)	29.21 (16.78)	34.98 (13.4)	0.865	0.917	33.42 (17.17)	34.97 (13.3)	0.416	
PSAD	0.25 (0.18)	0.18 (0.09)	0.49 (0.51)	0.048	0.941	0.21 (0.12)	0.23 (0.22)	0.386	
Free/total PSA ratio	0.16 (0.07)	0.17 (0.10)	0.12 (0.05)	0.889	0.037	0.09 (0.08)	0.15 (0.06)	0.529	

	PI-RASD 3 (20)			PI-RADS 4 (33)			PI-RADS 5 (27)		
	Concordance	Upgrade	P^b^	Concordance	Upgrade	P^b^	Concordance	Upgrade	P^b^
N	7 (35.0%)	9 (45.0%)		11 (33.3%)	13 (39.4%)		9 (33.3%)	8 (29.6%)	
Age	65.71 (4.04)	67.67 (5.47)	0.449	62.27 (5.86)	67.92 (6.15)	0.026	65.67 (6.92)	67.75 (6.94)	0.576
PSA	5.31 (2.46)	8.34 (2.73)	0.035	6.34 (2.49)	6.94 (3.29)	0.713	9.55 (5.47)	9.92 (6.97)	0.944
PV	31.52 (10.98)	30.03 (8.45)	0.769	31.98 (15.02)	35.72 (15.83)	0.55	31.22 (12.98)	29.13 (12.19)	0.790
PSAD	0.18 (0.09)	0.29 (0.11)	0.025	0.24 (0.14)	0.23 (0.13)	0.813	0.32 (0.16)	0.38 (0.28)	0.786
Free/total PSA ratio	0.14 (0.06)	0.19 (0.10)	0.182	0.10 (0.05)	0.14 (0.16)	0.100	0.12 (0.05)	0.15 (0.06)	0.308

^a^One-way ANOVA (Tukey’s HSD test).

^b^Student’s t-test.

Data are represented as mean (SD).

*PSA* prostate-specific antigen; *PV* prostate volume; *PSAD* prostate specific antigen density; *PI-RADS* Prostate Imaging Reporting and Data System.

## Discussion

Owing to the development of imaging techniques such as mpMRI, studies to improve the rate and accuracy of PCa diagnosis are being actively conducted^9,12,20^. A combination of targeted and systematic biopsies reportedly increases the diagnosis rate for PCa^21^. However, simple increase in PCa detection rate is associated with the overtreatment of indolent insignificant PCa or undertreatment of significant PCa. To prevent over- or under-treatment, the classification of cancer risk is essential, prostate biopsy plays a key role as a predictor of risk classification^17^. Based on strong evidence, targeted and systematic biopsies with MRI, and risk predictive models for PCa have been proposed for precise and individualized treatment^22^. In PCa, the biopsy result is used both for diagnosis according to the presence or absence of cancer and for cancer risk stratification. Thus, for accurate and reliable risk prediction for PCa, the reliability of biopsy results from prostatectomy specimens should be evaluated.

However, transrectal biopsy, which is the current standard, is associated with missed diagnosis and has an inconsistency of > 30%^6^. In a comparison study between prostate biopsy and prostatectomy specimen, targeted biopsy was associated with 30.9% of the upgraded grade group. In addition, 12-core systematic extended extant biopsy was associated with 41.6% of the upgrading grade group and combined targeted and systematic biopsy to 14.4% of the upgrading grade group^23^. In addition, in the present study, even a combination of targeted and systematic biopsy (bpMRI-US transperineal FTSB) showed 58.7% consistency, i.e., it was highly inconsistent in 41.3% of patients who had MRI-visible lesions, showing low reliability for risk classification.

This inconsistency of targeted and systematic biopsies are caused by several factors. Regardless of the presence of MRI-visible lesions, the grade group varies depending on where the biopsy is performed in the MRI-visible lesions, which is an inherent limitation of biopsy as a diagnostic tool in PCa. In addition, this inconsistency is associated with the multifocal nature of PCa. Up to 90% of whole-mount specimens have multifocality^7^, which is in agreement with the 81.2% multifocality reported in the present study. Furthermore, it has reported inter-observer variability in GrGp of the PCa grade group between pathologists^24,25^. Therefore, in the present study, biopsy and final specimen results were all reviewed lesion-by-lesion by a single pathologist to eliminate inter-observer variability and provide reliable comparison.

To determine whether PCa risk stratification could be estimated using prostate biopsy, it is necessary to identify the index lesion which can determine cancer risk and behavior, growth, cellular proliferation, and progression of cancers^7,8^. The ability to identified the location of index lesions will allow for accurate PCa risk stratification using targeted biopsy only, which is an ideal prostate biopsy that has also been performed for other cancers.

However, access to the index lesion with MRI is still incomplete. However, access to the index lesion using MRI is still imperfect. In a comparison study between preoperative mpMRI and prostatectomy specimens, mpMRI detected 45% of all lesions and 80% of high-grade tumors, but missed at least one csPCa foci in 34% of patients, including 45% of patients with multifocal lesions (81% of GS 6 tumors and 90% of tumors < 5 mm) ^26^. In addition, the area of the actual PCa was underestimated in the prostatectomy specimen than in the MRI-visible lesions because some regions of PCa were not visible even in the MRI-visible lesions^12^. Several factors influence visibility on MRI and the accuracy of volume assessment by tumor density, tumor size, ISUP grade, and location (i.e., intermediate density, diameter less than 10 mm, heterogeneous tumor morphology, and localization in the transitional zone) ^27,28^. Greater detection of csPCa and lower detection of clinically insignificant PCa are the primary benefits of adopting MRI^9^. The true clinical significance of these missed tumor foci that are invisible on MRI remains uncertain.

The current study is based on a contrast-free protocol for bpMRI instead of mpMRI. Although a contrast-free protocol using bpMRI is a relatively novel topic in prostate cancer, several studies have already evaluated the performance of mpMRI and bpMRI^29–31^. In a systematic review, bpMRI offered test accuracies comparable to those of mpMRI; BpMRI vs. mpMRI sensitivity, 0.82 versus 0.89 (*p* = 0.39); specificity, 0.79 versus 0.74 (*p* = 0.53).^29^ AUC for PCa staging was not significantly improved (mpMRI, AUC = 0.73 vs. bpMRI, AUC = 0.76) by DCE sequence with contrast^30^. Furthermore, bpMRI is a contrast-free technique, rapid (~ 15 min), and simpler, while sufficiently retaining the diagnostic value of mpMRI^31^. Owing to these advantages of a contrast-free protocol, bpMRI was adopted in this study instead of mpMRI and we already reported the diagnostic performance and risk calculation for csPCa based on bpMRI^19^.

In the present study, by comparing prostatectomy specimen and MRI, 32.5% of clinically significant prostate cancers (GrGp ≥ 2) were found in MRI-invisible lesions and 6.3% of index lesions were found in MRI-invisible lesions. Thus, even in the era of targeted biopsy with MRI, targeted biopsy alone is insufficient to predict PCa risk classification, systematic biopsy cannot be omitted because systematic biopsy plays a role in detecting 6.3% of index lesions in MRI-invisible lesions. Moreover, the result of a combination of targeted and systematic biopsy with MRI-visible lesions marked with ≥ 3 in PI-RADS could be upgraded to grade group in 35.0 and 71.4% of insignificant to a significant cancer. Even combined targeted biopsy with transperineal template systematic biopsy, which is considered the most reliable, matched only 58.7 and 35.0% were upgraded to prostatectomy specimen. Thus, it is needed to doubt the reliability of diagnosed insignificant cancers according to biopsy, which can be formed in the visible area in MRI. Moreover, when urologist decide the treatment options such as active surveillance for indolent low-risk localized PCa, a comprehensive interpretation of clinical parameters, MRI, and biopsy results is necessary for PCa risk classification. When the prostatectomy specimens were compared with biopsy results, age, PSA, and PSAD tended to be higher in the upgraded group than in the concordant group. Specifically, in the PIRADS 3 group, PSA and PSAD were significantly higher in the upgraded group. Management of PI-RADS category 3 lesions is an area of uncertainty^32^. PSAD has been proposed to improve the predictive value of csPCa in combination with the PI-RADS category. The csPCa-free survival was significantly different in men with a suspicious MRI and subsequent negative targeted prostate biopsy, and a threshold of 0.15 PSAD was associated with a significant risk of csPCa^33^. Furthermore, PSAD (> 0.15) improves specificity and PPV in men with PI-RADS category 3 and contributes to improved management of csPCa^34^. Thus, even if the biopsy result is insignificant cancer, precise and individualized strategies for PCa treatment should be established considering that if the PSA, PSAD, and PI-RADS scores are high, real risk classification may be higher than that of the grade group diagnosed by biopsy.

The ultimate aim of this study was to provide risk stratification based on the PI-RADS score, clinical parameters, and results of targeted and systematic biopsies. The limitation of this study is that the included number of patients is insufficient, a cut-off value for result interpretation and risk stratification could not be provided. Although PSA and PSAD showed a tendency to be higher in PI-RADS 4 and 5, and it showed statistical significance in a specific group of PI-RADS 3. Therefore, comparative analysis of a larger number of specimens is needed to provide a criterion of risk classification for precise and individualized strategies for PCa treatment.

## Conclusion

The results of prostate biopsy were associated with inconsistency and underestimation compared to prostatectomy specimens. For precise and individualized treatment strategies in patients who had MRI-visible lesions, careful interpretation of MRI, clinical parameters, and results of biopsy which should not omit systematic biopsy, is required.

### Data availability

All data generated or analysed during this study are included in this article and its supplementary information files. The datasets used and/or analysed during the current study available from the corresponding author on reasonable request.

## Abbreviations

bpMRI: Bi-parametric MRI
BpMRI–US FTSB: Bi-parametric MRI-Ultrasound fusion (transperineal) targeted and template systematic biopsy
csPCa: Clinically significant prostate cancer
mpMRI: Multi-parametric MRI
MRI: Magnetic resonance imaging
PCa: Prostate cancer
PI-RADS: Prostate Imaging–Reporting and Data System
PSA: Prostate specific antigen
PSAD: Prostate specific antigen density
PV: Prostate volume
RARP: Robot-assisted radical prostatectomy
ROI: Regions of interest
TRUS: Transrectal ultrasound guided
US: Ultrasound
